# Could ERAS protocols be integrated into the national Standards for Quality in Healthcare in Türkiye?

**DOI:** 10.55730/1300-0144.5975

**Published:** 2025-01-04

**Authors:** Mine GÜRSAÇ ÇELİK, Adem AZ, Mehmet ÇETİN, Neslihan ALKIŞ

**Affiliations:** 1 Department of Anesthesiology and Reanimation, Haseki Training and Research Hospital, University of Health Sciences, İstanbul, Turkiye; 2 Department of Emergency Medicine, Haseki Training and Research Hospital, University of Health Sciences, İstanbul, Turkiye; 3 Department of Internal Medicine, Haseki Training and Research Hospital, University of Health Sciences, İstanbul, Turkiye; 4 Department of Anesthesiology and Reanimation, Faculty of Medicine, Ankara University, Ankara, Turkiye

**Dear Editor**,

We are writing to propose the integration of Enhanced Recovery After Surgery (ERAS) protocols into the Standards for Quality in Healthcare (SQH; Turkish: *Sağlıkta Kalite Standartları*) guidelines established by the Ministry of Health of Türkiye. Established in 2003 as part of the Health Transformation Program, the SQH guidelines have served as a comprehensive framework to improve healthcare quality nationwide. Over the years, these standards have evolved to incorporate various dimensions of healthcare delivery. Specifically, the SQH guidelines aim to enhance patient safety, improve the quality of healthcare delivery, and increase efficiency. They outline regulations targeting patient care, medication management, infection prevention, disinfection and sterilization, operating room protocols, and intensive care services. However, while the SQH have significantly advanced healthcare standards, they lack specific, detailed protocols addressing enhanced recovery pathways such as those defined by ERAS.

ERAS constitutes a multidisciplinary strategy designed to accelerate postoperative recovery and enhance patient outcomes through evidence-based practices [1]. The ERAS protocols, originally developed in the late 1990s, have gained global recognition for their success in reducing surgical complications, shortening hospital stays, and improving patient satisfaction. Studies conducted in the United Kingdom demonstrated that ERAS implementation in colorectal surgery reduced the average length of hospital stay by 2–3 days and lowered complication rates by approximately 30% [2]. Similarly, hospitals in Denmark have reported a 20% improvement in patient satisfaction scores following the integration of ERAS into orthopedic surgical care [3]. Moreover, prehabilitation programs emphasize structured exercise and nutritional optimization [4]. However, key elements of ERAS are either insufficiently addressed or absent in the SQH framework. Incorporating ERAS protocols into the SQH framework will significantly enhance postoperative care and patient outcomes while positioning Türkiye as a leader in adopting modern, evidence-based practices.

Below, we present several recommendations for integrating ERAS protocols within the current SQH framework.

1. **Alignment of SQH with ERAS protocols:** The SQH guidelines encompass quality management at the preoperative, intraoperative, and postoperative stages, which is consistent with ERAS elements such as preoperative preparation, postoperative pain management, nutrition, and mobilization. The integration of ERAS nutrition and mobilization protocols within the “patient care processes” section of the SQH could facilitate the standardization of enhanced recovery practices across facilities. The SQH could incorporate specific metrics such as ensuring that all patients receive carbohydrate-loading drinks prior to surgery or implementing protocols for mobilization within 24 h after surgery [4].
2. **Teamwork and coordination:** The ERAS protocols require coordination among surgeons, nurses, anesthesiologists, dietitians, and other healthcare professionals, reflecting the multidisciplinary teamwork that the SQH guidelines mandate for patient safety. Aligning SQH team management standards with ERAS requirements could enhance collaborative care and facilitate ERAS implementation. Practical steps could include the formation of multidisciplinary ERAS committees within hospitals to oversee protocol adoption and ensure consistent communication among team members [4,5].
3. **Patient education protocols:** The SQH guidelines emphasize informed consent and patient education, which are also integral to ERAS. Informing patients about pre- and postoperative expectations and procedures is essential for ERAS success. The SQH for patient rights and education could be expanded to cover ERAS-specific informational needs, enhancing patient engagement and understanding. Specific updates could include the development of standardized patient education materials that outline ERAS protocols, such as instructional videos or brochures for preoperative preparation [4].
4. **Performance monitoring and improvement:** The SQH guidelines incorporate quality improvement and performance monitoring. ERAS focuses on outcome-based performance, allowing hospitals to track patient recovery data under SQH guidelines. By aligning SQH data collection and evaluation standards with ERAS protocols, hospitals could continuously improve postoperative outcomes. ERAS-specific key performance indicators can be integrated into existing monitoring frameworks, such as monitoring early mobilization rates or adherence to nutritional guidelines [5].
5. **Personnel training:** Continuous training and professional development are central to the SQH. Regular education and training are also critical for the successful application of ERAS protocols. By updating the SQH guidelines on “staff training” to include ERAS guidelines, healthcare staff can be effectively trained to deliver ERAS-aligned care. This training could involve the creation of ERAS training modules tailored to different professional roles, such as workshops for surgeons on minimally invasive techniques or seminars for nurses on enhanced postoperative care.
6. **Challenges and future outlook:** Integrating ERAS protocols into the SQH framework constitutes a transformative opportunity; however, challenges such as resistance to change, resource constraints, and the necessity of widespread training must be acknowledged. Addressing these issues requires proactive strategies, including stakeholder engagement, phased implementation, and the allocation of sufficient resources for training and infrastructure development. Successful pilot programs in select hospitals could serve as models for nationwide implementation.

In conclusion, incorporating ERAS protocols into the SQH framework will not only enhance the quality of postoperative care but will also elevate Türkiye’s healthcare system to meet international standards. By focusing on evidence-based practices, patient-centered care, and continuous improvement, this integration has the potential to significantly improve patient outcomes and set a benchmark for modern medical advancements in the region. The urgency to act lies in the growing demand for effective, efficient, and patient-focused surgical care. Adopting ERAS protocols within the SQH framework represents a vital step forward in achieving these goals. We thank our readers for considering this proposal for a more integrated approach to postoperative care standards in Türkiye.
